# Knowledge, attitudes, and practices regarding cataract and diabetic retinopathy among middle-aged population and older adults in a rural area at eastern China

**DOI:** 10.3389/fpubh.2026.1888283

**Published:** 2026-09-01

**Authors:** Junmei Pan, Yiqing Zhang, Ran Wu, Yan Cui, Hongling Yang

**Affiliations:** 1 Department of Ophthalmology, Qilu Hospital of Shandong University, Shandong University, Jinan, Shandong, China; 2 School of Medicine, Shandong University, Jinan, Shandong, China; 3 Dongda Hospital of Shanxian, Heze, Shandong, China

**Keywords:** attitudes, cataract, diabetic retinopathy, health knowledge, practice

## Abstract

**Background:**

Cataract and diabetic retinopathy (DR) are the leading causes of visual impairment and blindness among middle-aged and older adults. This study aimed to investigate the knowledge, attitudes, and practices (KAP) of cataract and DR among middle-aged and older adults residing in a rural region of Eastern China.

**Methods:**

This cross-sectional study was carried out from March to September 2024, targeting middle-aged and older adults aged 40 to 80 years residing in the rural region of Huanggang town, Shanxian county, Shandong province. Participants provided demographic data, as well as information on their knowledge, attitudes, and practices, through a self-administered questionnaire.

**Results:**

A total of 460 questionnaire responses were incorporated into the analysis. The mean scores for knowledge regarding cataracts, knowledge concerning DR, attitude, and practice were 14.54 ± 8.14 (possible range: 0–28), 23.84 ± 17.64 (possible range: 0–46), 37.02 ± 8.09 (possible range: 9–45), and 26.02 ± 5.33 (possible range: 8–40), respectively. The analysis indicated that knowledge of DR was positively associated with attitude (*β* = 0.565, 95% CI: 0.454 to 0.675, *p* < 0.001), whereas knowledge of cataract was positively associated with practice (*β* = 0.428, 95% CI: 0.298 to 0.558, *p* < 0.001).

**Conclusion:**

This study revealed that while the knowledge and practices regarding cataracts and DR among the rural middle-aged and older adults population in the area remain inadequate, there is a prevalent desire for increased support and education. Consequently, it is imperative for relevant departments and healthcare organizations to implement measures aimed at educating both patients and the general public about cataracts and DR.

## Introduction

Currently, blinding eye diseases pose a substantial challenge in the realm of global public health (1). Among these, cataracts and diabetic retinopathy (DR) are prevalent conditions, with their incidence rising on a global scale (1, 2). Cataracts represent a primary cause of vision impairment, responsible for more than 50% of global blindness cases (3). By 2020, the prevalence of cataracts had increased with population aging, particularly in remote and underserved regions of low- and middle-income countries. Surgical intervention remains the only effective treatment for cataracts; however, costs vary across providers in China (4). DR predominantly impacts the microvasculature of the retina, leading to diminished visual acuity and affecting approximately one-third of individuals with diabetes. Of these individuals, over 10% may experience irreversible vision loss (5, 6). DR is currently recognized as the leading cause of blindness and visual impairment among middle-aged and older adults (5, 7). A study conducted in 2021 reported that the global prevalence of DR among individuals with diabetes is 22.27%, with a comparable prevalence rate of 22.4% observed in China (8). The onset and progression of DR not only result in blindness but also significantly impair patients’ quality of life and lead to substantial socio-economic losses (9).

Addressing the serious issue of blinding eye diseases requires proactive behavior from both the public and patients. The public should increase awareness, understand risks, adopt good eye care habits, and learn preventive measures. Patients need to manage their conditions, cooperate with doctors, and undergo regular checkups for early detection and treatment. Additionally, engaging in rehabilitation can enhance patients’ quality of life. By working together, the public and patients can significantly reduce the incidence and impact of these diseases, easing the societal and familial health burden (1). The Knowledge, Attitude, and Practice (KAP) Model is a well-established theoretical framework for influencing human health behavior, characterized by the interrelationship among the three components: knowledge, attitude, and practice (10). This model is instrumental in assessing the awareness levels and behavioral patterns of specific populations concerning particular health issues. In this study, the KAP survey was chosen for implementation among the rural middle-aged and senior population, as this demographic is more vulnerable to health risks (11). Contributing factors such as potential deficiencies in medical information, limited resources, lower educational attainment, and reduced socioeconomic status prevalent in rural areas may exacerbate this group’s lack of knowledge regarding eye diseases or lead to an underestimation of their prevalence and severity (11).

The KAP survey methodology serves as an effective tool for informing disease assessment and intervention strategies. Numerous prior KAP studies have demonstrated significant disparities in the knowledge, attitudes, and practices related to cataract and DR among populations across different global regions (12–19). These disparities are predominantly influenced by factors such as economic status, availability of medical resources, and cultural background. Nevertheless, there is a paucity of data concerning rural middle-aged population and older adults in China. A limited number of studies have indicated low levels of knowledge regarding cataract and DR among middle-aged population and older adults in rural areas of western China (11, 19). In these regions, the level of knowledge is closely linked to attitudes and practices and is associated with factors such as educational attainment and economic income. Investigating the knowledge, attitudes, and practices concerning cataract and DR among the middle-aged population and older adults in rural China can address existing research gaps and provide a foundation for developing targeted health education and intervention programs. Therefore, this study aims to assess the KAP regarding cataract and DR among middle-aged and older adults residing in a rural region of Eastern China. By conducting a KAP survey, the research seeks to establish a foundation for the development of more effective prevention and intervention strategies. Gaining insights into the current awareness levels of this population will facilitate the optimization of medical resource allocation and enhance their capacity to address and manage ocular health issues, thereby contributing to the improvement of overall eye health in rural areas.

## Materials and methods

### Study design and participants

This cross-sectional study was conducted between March and September 2024 and included permanent residents aged 40 to 80 years within a rural region (Huanggang Town, Shanxian County) in Shandong Province, China. The inclusion criteria were: (1) permanent residents aged 40 to 80 years in rural areas of Shanxian County, and (2) individuals who provided informed consent and expressed willingness to participate in the study. The exclusion criteria included: (1) individuals unable to communicate, (2) local residents who resided outside the area for extended periods. Participants were recruited through community health workers who distributed the QR code survey link to eligible residents during routine health check-ups and community gatherings.

### Sample size

The sample size was calculated using Cochran’s formula for cross-sectional studies:

$$n=\frac{Z^{2}\times p\times (1-p)}{E^{2}}$$

where *n* is the required sample size; *Z* is the *z*-value corresponding to the desired confidence level (for a 95% confidence level, *Z* = 1.96); *p* is the estimated proportion of the population (commonly assumed to be 0.5 when unknown); *E* is the margin of error (typically set at 0.05). For a 95% confidence level, with *p* = 0.5 and *E* = 0.05, the formula calculates as: *n* = (1.96^2^ × 0.5 × (1 – 0.5))/0.05^2^ = 384.16. Therefore, at least 384 valid questionnaires were required.

### Questionnaire

The design of the questionnaire was based on literature sources (20, 21), the study titled “Survey and Intervention Research on the Knowledge, Attitudes, and Practices of Diabetic Patients Regarding DR” by Wu Yunyan, as well as relevant guidelines and clinical experience in the field. The questionnaire items were reviewed by two experts (one in ophthalmology and one in public health, both with over 20 years of experience) in the field to assess their accuracy and content relevance. A small-scale pilot test (28 samples) was conducted, with a reliability score of 0.957.

The knowledge dimension was divided into questions specific to cataract (14 items in total, scoring, “understand” = 2 points, “partially understand” = 1 point, and “do not understand” = 0 points) and DR-related questions (24 items in total, scoring, “correct/yes” = 2 point, “incorrect/no” or “unclear” = 0 points). The attitude dimension included 9 items, with items 1–5 and 9 adopting a five-point Likert scale ranging from “strongly agree” (5 points) to “strongly disagree” (1 point), yielding a total score range of 9–45 points. The practice dimension consisted of 8 items, also using a five-point Likert scale, with a total score range of 8–40 points. The overall scores for patients’ knowledge, attitudes, and practices were classified according to a modified Bloom’s criteria cutoff point. Respondents achieving scores between 80–100% were deemed to possess good knowledge, a positive attitude, and appropriate practices. Scores ranging from 60–79% were categorized as moderate, while scores below 60% were considered indicative of poor knowledge, a negative attitude, and inappropriate practices (22).

Data collection was facilitated through a questionnaire distributed to participants via a QR code generated by the “Questionnaire Star” platform. Quality control measures included IP address restrictions to prevent duplicate submissions, mandatory completion of all questions, and logical consistency checks to identify invalid responses. To ensure data quality, we implemented several control measures: (1) setting a minimum completion time threshold to identify rushed responses, (2) incorporating attention-check questions to detect inattentive respondents, (3) using logic checks to identify inconsistent responses, and (4) having trained field workers available to assist participants with technical difficulties or comprehension issues (23). Questionnaires were excluded if participants did not meet the inclusion criteria, provided conflicting responses, completed the questionnaire in less than 80 s, or submitted incomplete responses.

### Statistical methods

Normality of continuous variables was assessed using the Shapiro–Wilk test. Normally distributed continuous variables were presented as the mean and standard deviation (SD) and compared using t-tests or analysis of variance (ANOVA). Continuous data with a skewed distribution were described using the median (P25, P75), and compared by Wilcoxon-Mann–Whitney or Kruskal-Wallis tests. Correlation analysis of scores within each dimension was conducted using Spearman correlation coefficients for data not meeting normality assumptions.

Utilizing the Knowledge, Attitude, and Practice (KAP) theoretical framework, a structural equation model (SEM) was employed to assess whether attitude serves as a mediator in the relationship between knowledge and practice. Both indirect and direct effects were computed and compared. Model fit was evaluated using RMSEA, SRMR, CMIN/DF, IFI, TLI, and CFI. Confirmatory factor analysis (CFA) was conducted using data collected from the formal survey to evaluate the construct validity of the questionnaire. Because practice represents the behavioral component of the KAP framework and is directly related to disease prevention, screening, and healthcare-seeking behavior, the practice score was selected as the dependent variable. Univariate linear regression analyses were first performed to evaluate the associations of demographic characteristics, disease-related characteristics, knowledge scores, and attitude scores with practice scores. Variables identified through the univariate analyses, together with clinically relevant factors, were subsequently considered in the multivariable linear regression model. Regression coefficients (*β*), 95% confidence intervals (CIs), and *p*-values were reported. All statistical analyses were executed using Stata version 18.0, and a two-sided *p* < 0.05 was considered statistically significant.

## Results

### Characteristics of the participants

A total of 590 questionnaires were initially collected. Of these, 130 were excluded because the participants did not meet the inclusion criteria (*n* = 51), provided conflicting responses (*n* = 36), completed the questionnaire in less than 80 s (*n* = 33), or submitted incomplete responses (*n* = 10). Consequently, 460 valid questionnaires were included in the final analysis. For all collected questionnaires (*n* = 460), the overall Cronbach’s *α* was 0.893, and the Kaiser-Meyer-Olkin (KMO) measure of sampling adequacy was 0.915 (*p* < 0.001), indicating good reliability and validity.

The 460 participants consisted of 175 males (38.0%) and 285 females (62.0%), with the highest participation rate in the 40–50 age group (52.8%), followed by the 51–60 age group (31.7%). The education of the study subjects varied, with 116 subjects (25.2%) having tertiary education or above, 190 subjects (41.3%) having attended middle/high school/vocational school, and 154 subjects (33.5%) having only elementary school education or below. Their economic level was generally low, with 311 subjects (67.6%) having a per capita monthly household income of less than 5,000 yuan. In addition, 45 (9.8%) had diabetes mellitus, of which 13 (2.8%) had been diagnosed with DR. 50 (10.9%) had cataract, of which 34 (7.4%) had senile cataract (Table 1).

**Table 1:** Participant characteristics and scores for knowledge of cataract, knowledge of diabetic retinopathy, attitude, and practice.

Characteristics	*N* (%)	Knowledge on cataract	*p*	Knowledge on DR	*p*	Attitude	*p*	Practice	*p*
		Mean ± SD		Mean ± SD		Mean ± SD		Mean ± SD	
Total score	460 (100.0)	14.54 ± 8.14		23.84 ± 17.64		37.02 ± 8.09		26.02 ± 5.33	
Age, years			0.003		0.025		0.207		<0.001
≤50	243 (52.8)	15.74 ± 8.46		25.57 ± 18.23		37.61 ± 7.75		26.99 ± 5.28	
51–60	146 (31.7)	13.58 ± 7.85		23.39 ± 17.70		36.50 ± 8.22		24.84 ± 5.38	
>60	71 (15.4)	12.41 ± 6.92		18.83 ± 14.34		36.07 ± 8.89		25.13 ± 4.81	
Gender			0.823		0.341		0.128		0.004
Male	175 (38.0)	14.31 ± 7.96		22.83 ± 17.27		36.43 ± 8.03		25.14 ± 5.30	
Female	285 (62.0)	14.68 ± 8.26		24.45 ± 17.86		37.39 ± 8.12		26.56 ± 5.29	
Education			<0.001		<0.001		0.241		0.285
Primary school or below	154 (33.5)	12.31 ± 7.98		19.76 ± 17.02		36.33 ± 8.69		25.66 ± 4.77	
Secondary school/High school/Vocational school	190 (41.3)	15.06 ± 8.14		24.19 ± 17.89		36.88 ± 8.19		25.93 ± 5.79	
College or above	116 (25.2)	16.64 ± 7.69		28.66 ± 16.87		38.17 ± 6.97		26.66 ± 5.25	
Monthly income, CNY			0.056		0.107		0.756		0.846
<5,000	311 (67.6)	13.90 ± 8.17		22.83 ± 17.61		37.09 ± 7.87		25.96 ± 5.33	
5,000–10,000	106 (23.0)	15.81 ± 7.99		24.96 ± 17.08		36.88 ± 8.14		26.08 ± 5.27	
>10,000	43 (9.3)	16.05 ± 7.85		28.35 ± 18.68		36.86 ± 9.60		26.28 ± 5.63	
Diabetes			0.008		0.348		0.995		0.104
Yes	45 (9.8)	11.58 ± 5.84		21.84 ± 13.37		37.22 ± 8.21		24.80 ± 4.80	
No	415 (90.2)	14.86 ± 8.29		24.05 ± 18.04		37.00 ± 8.09		26.15 ± 5.38	
Family history of diabetes			<0.001		0.001		0.059		0.013
Yes	45 (9.8)	13.09 ± 6.73		24.04 ± 13.77		38.82 ± 6.21		25.29 ± 4.70	
No	365 (79.3)	15.28 ± 8.19		25.03 ± 18.01		37.09 ± 8.18		26.35 ± 5.49	
Unsure	50 (10.9)	10.46 ± 7.65		14.92 ± 15.55		34.88 ± 8.62		24.24 ± 4.23	
Cataracts			0.310		0.599		0.847		0.116
Yes	50 (10.9)	13.18 ± 5.51		23.14 ± 12.87		38.30 ± 4.55		24.86 ± 4.40	
No	410 (89.1)	14.70 ± 8.39		23.92 ± 18.15		36.87 ± 8.41		26.16 ± 5.42	
Family history of cataracts			<0.001		<0.001		0.834		<0.001
Yes	27 (5.9)	14.37 ± 7.46		22.00 ± 17.15		38.44 ± 5.17		25.26 ± 4.90	
No	347 (75.4)	15.46 ± 8.25		25.65 ± 18.20		37.04 ± 8.13		26.64 ± 5.44	
Unsure	86 (18.7)	10.90 ± 6.84		17.12 ± 13.50		36.51 ± 8.69		23.74 ± 4.31	
BMI, kg/m^2^			0.121		0.372		0.616		0.11
<24	216 (47.0)	13.83 ± 8.14		23.08 ± 17.78		36.93 ± 8.06		26.39 ± 5.22	
≥24	244 (53.0)	15.16 ± 8.10		24.51 ± 17.52		37.11 ± 8.14		25.69 ± 5.42	
Blood glucose within the normal range			<0.001		<0.001		<0.001		<0.001
Always	207 (45.0)	16.85 ± 8.33		29.08 ± 17.18		38.44 ± 7.49		27.88 ± 5.42	
Other	253 (55.0)	12.65 ± 7.48		19.55 ± 16.86		35.86 ± 8.38		24.50 ± 4.76	
Blood lipid level within the normal range			<0.001		<0.001		0.051		<0.001
Always	171 (37.2)	17.22 ± 8.34		27.53 ± 17.78		37.67 ± 8.12		28.04 ± 5.28	
Other	289 (62.8)	12.95 ± 7.60		21.65 ± 17.22		36.64 ± 8.06		24.83 ± 5.00	

### Knowledge, attitude, and practice

The mean scores regarding knowledge (cataract), knowledge (DR), attitude and practice were (14.54 ± 8.14), (23.84 ± 17.64), (37.02 ± 8.09) and (26.02 ± 5.33) respectively. In the analysis of cataract knowledge scores, significant differences were observed in relation to age, education, diabetes prevalence, family history of diabetes and cataract, as well as blood glucose and lipid levels, compared to the mean knowledge scores of the participants (*p* < 0.05). Similarly, in the evaluation of DR knowledge scores, age, education, family history of diabetes and cataract, and blood glucose and lipid levels also demonstrated significant deviations from the mean knowledge scores of the participants (*p* < 0.05). Furthermore, blood glucose levels exhibited significant differences (*p* < 0.05) with respect to the participants’ attitude scores toward cataract and DR. Additionally, age, gender, family history of diabetes and cataract, and blood glucose and lipid levels were significantly correlated (*p* < 0.05) with the participants’ practice scores (Table 1). The model fit indices from the confirmatory factor analysis are presented in Supplementary Table 1. The results showed that RMSEA (0.080), CMIN/DF (3.914), IFI (0.856), TLI (0.847), and CFI (0.856) were acceptable, while the SRMR value (0.1046) exceeded the threshold of 0.08, indicating an acceptable but suboptimal model fit.

#### Knowledge on cataract

The data presented in Table 2 illustrate the participants’ level of knowledge regarding cataracts. Overall, 24.6% of participants were aware of the diagnostic procedures for cataracts, while 24.8% understood the classification of cataracts as either congenital or acquired, with senile cataracts being the most prevalent type of acquired cataracts. Additionally, 27.8% recognized that the most common symptom of cataracts is a gradual and painless loss of vision, accompanied by other symptoms such as blurred vision and loss of color vision. Furthermore, 27% were aware that early-stage cataracts are not easily detectable and can be mistaken for presbyopia or visual fatigue. A notable 43.3% of respondents knew that cataracts may be associated with advanced age, glaucoma, high myopia, and metabolic diseases such as diabetes mellitus. Moreover, 31.7% understood that untreated cataracts could lead to secondary glaucoma, visual impairment, and even blindness. Finally, 35.2% were aware that surgery is the definitive treatment for cataracts. However, only 36.3% of individuals are aware that patients with both diabetes and cataracts must stabilize their blood glucose levels and undergo a comprehensive funduscopic examination prior to surgical intervention. Furthermore, 33.7% are cognizant of the potential complications associated with cataract surgery, such as infections, inflammatory reactions, and dry eye syndromes. Additionally, 30% understand that the majority of patients can achieve a corrected distance vision of 1.0 or better following cataract surgery.

**Table 2:** Response to knowledge on cataract.

Knowledge on cataract	Knowledgeable	Heard of it	Unaware
1. Are you aware of the diagnostic methods for cataracts?	113 (24.6%)	219 (47.6%)	128 (27.8%)
2. Cataracts can be classified as congenital or acquired, with senile cataracts being the most common type among acquired cataracts.	114 (24.8%)	203 (44.1%)	143 (31.1%)
3. The most typical symptom of cataracts is a gradual, painless decline in vision. Other symptoms include blurred vision and loss of color vision.	128 (27.8%)	209 (45.4%)	123 (26.7%)
4. Early-stage cataracts have inconspicuous symptoms and can be easily confused with presbyopia or visual fatigue.	124 (27%)	194 (42.2%)	142 (30.9%)
5. Are you aware that the occurrence of cataracts may be related to the following factors?			
Advanced age, glaucoma, high myopia, metabolic diseases (e.g., diabetes)	199 (43.3%)	132 (28.7%)	129 (28%)
Intense light exposure, radiation exposure, high temperature working environment	185 (40.2%)	123 (26.7%)	152 (33%)
History of ocular trauma, inflammation, or surgery	184 (40%)	131 (28.5%)	145 (31.5%)
Poor lifestyle habits (e.g., smoking, excessive alcohol consumption, obesity)	174 (37.8%)	132 (28.7%)	154 (33.5%)
Long-term use of medications such as glucocorticoids or miotic agents	157 (34.1%)	113 (24.6%)	190 (41.3%)
6. If cataracts are not treated promptly, secondary glaucoma, visual impairment, or even blindness may occur.	146 (31.7%)	206 (44.8%)	108 (23.5%)
7. Treatment for cataracts includes pharmacological and surgical interventions, with surgery being the primary treatment option.	162 (35.2%)	217 (47.2%)	81 (17.6%)
8. If the patient has both diabetes and cataracts, it is necessary to stabilize blood glucose levels and perform a comprehensive fundus examination before surgical treatment.	167 (36.3%)	180 (39.1%)	113 (24.6%)
9. Surgical treatment of cataracts may lead to complications such as infection, inflammatory reactions, and dry eye syndrome.	155 (33.7%)	190 (41.3%)	115 (25%)
10. Post-surgical outcomes indicate that most patients achieve a corrected distance visual acuity of 1.0 or higher.	138 (30%)	147 (32%)	175 (38%)

#### Knowledge on DR

The data presented in Table 3 illustrates the survey results regarding participants’ knowledge of DR. Overall, only 29.3% of respondents reported understanding the diagnostic criteria for DR, while 42.6% recognized it as a complication of diabetes mellitus. Additionally, 44.3% were aware that DR can develop even with optimal glycemic control. Approximately 50% of participants understood the factors and symptoms associated with DR development, and 37.2% were knowledgeable about the classification of DR into non-proliferative and proliferative stages based on severity. Furthermore, 46.3% acknowledged the necessity of funduscopic examinations and regular follow-ups for diabetic patients. Awareness of pharmacologic or surgical treatment options for DR was reported by 48.5% of respondents, while 43.9% were well-informed about non-pharmacologic management strategies, such as diet and exercise.

**Table 3:** Response to knowledge on DR.

Knowledge on DR	Participants’ responses		
1. Do you understand the diagnostic criteria for diabetic retinopathy (DR)?	Yes	No	
	135 (29.3%)	325 (70.7%)	
	Very knowledgeable	Heard of It	Unaware
2. DR is one of the complications of diabetes.	196 (42.6%)	47 (10.2%)	217 (47.2%)
3. Patients with diabetes may develop DR even if their blood glucose levels are well controlled.	204 (44.3%)	42 (9.1%)	214 (46.5%)
4. Are you aware that the onset of DR may be related to the following factors?			
Age at diabetes onset	250 (54.3%)	26 (5.7%)	184 (40%)
Fasting blood glucose levels	257 (55.9%)	28 (6.1%)	175 (38%)
Glycated hemoglobin levels	246 (53.5%)	24 (5.2%)	190 (41.3%)
Hypertension	239 (52%)	26 (5.7%)	195 (42.4%)
Hyperlipidemia	247 (53.7%)	29 (6.3%)	184 (40%)
Smoking	220 (47.8%)	34 (7.4%)	206 (44.8%)
Poor treatment adherence	211 (45.9%)	32 (7%)	217 (47.2%)
Alcohol consumption	212 (46.1%)	37 (8%)	211 (45.9%)
Renal function	199 (43.3%)	31 (6.7%)	230 (50%)
5. Are you aware of the following symptoms of DR?			
Decreased vision	267 (58%)	24 (5.2%)	169 (36.7%)
Flashing lights or floaters	245 (53.3%)	32 (7%)	183 (39.8%)
Blurred vision (or distorted images)	252 (54.8%)	27 (5.9%)	181 (39.3%)
Visual field loss	204 (44.3%)	34 (7.4%)	222 (48.3%)
Double vision	187 (40.7%)	32 (7%)	241 (52.4%)
Darkening or fading of colors	199 (43.3%)	33 (7.2%)	228 (49.6%)
Eye pain	195 (42.4%)	39 (8.5%)	226 (49.1%)
Blindness	211 (45.9%)	28 (6.1%)	221 (48%)
6. Are you aware that DR can be classified into non-proliferative and proliferative forms based on severity?	171 (37.2%)	30 (6.5%)	259 (56.3%)
7. Are you aware that patients with diabetes need to undergo a fundus examination upon diagnosis and regularly receive follow-up assessments?	213 (46.3%)	25 (5.4%)	222 (48.3%)
8. Are you aware that DR can be treated with medications or surgical interventions?	223 (48.5%)	29 (6.3%)	208 (45.2%)
9. Are you aware that DR can also be managed through non-pharmacological means such as diet and exercise?	202 (43.9%)	41 (8.9%)	217 (47.2%)

#### Attitude

The data presented in Supplementary Table 2 illustrates the subjects’ attitudes toward cataracts and DR. No significant relationship was found between the mean scores of subjects’ attitudes and demographic factors such as age, gender, education, income, and disease history. However, a correlation was observed between blood glucose levels and attitudes. Specifically, 75.9% of participants believed that cataracts and DR affect vision and can lead to blindness in severe cases. Additionally, 75.2% of respondents believed that personal action is crucial in preventing and managing cataracts and DR. Furthermore, 76.7% emphasized the necessity of regular vision and funduscopic examinations, even if vision appears normal. Finally, 79.6% of subjects asserted that proper dietary control is essential for preventing or treating cataracts and DR. A total of 82% of respondents believed that modifying unhealthy lifestyle habits, such as smoking, alcohol consumption, and overeating, is crucial for the prevention or treatment of cataracts and DR. Additionally, 8.9% expressed concerns that normal vision might not be restored even after surgical intervention. Concerns about potential complications arising from surgery were noted by 3.9% of participants, while 9.8% were apprehensive about the risk of vision loss following surgery. In terms of the necessity for the prevention and management of cataracts and DR, 70.2% of respondents recognized the importance of regular fundus and vision examinations, 79.6% emphasized the need for education related to these diseases, 80.3% highlighted the importance of dietary and treatment guidance, and 79.8% advocated for health talks.

#### Practice

The data presented in Supplementary Table 3 illustrates the subjects’ evaluation of cataract and DR within the practice dimensions. Specifically, 59.4% of participants managed their blood glucose and lipid levels through dietary control, while 50% did so by taking medication. Additionally, 39% underwent regular vision and fundus examinations. A total of 50.4% successfully altered unhealthy lifestyle habits, such as smoking and drinking. Furthermore, 41.5% protected their eyes by wearing sunglasses or similar protective measures when outdoors. However, 46.4% reported experiencing frequent eye fatigue. Notably, 33% used medication without consulting a doctor, whereas 52.4% sought medical advice immediately upon experiencing eye symptoms.

### Correlation and interaction among KAP

**Figure 1:**
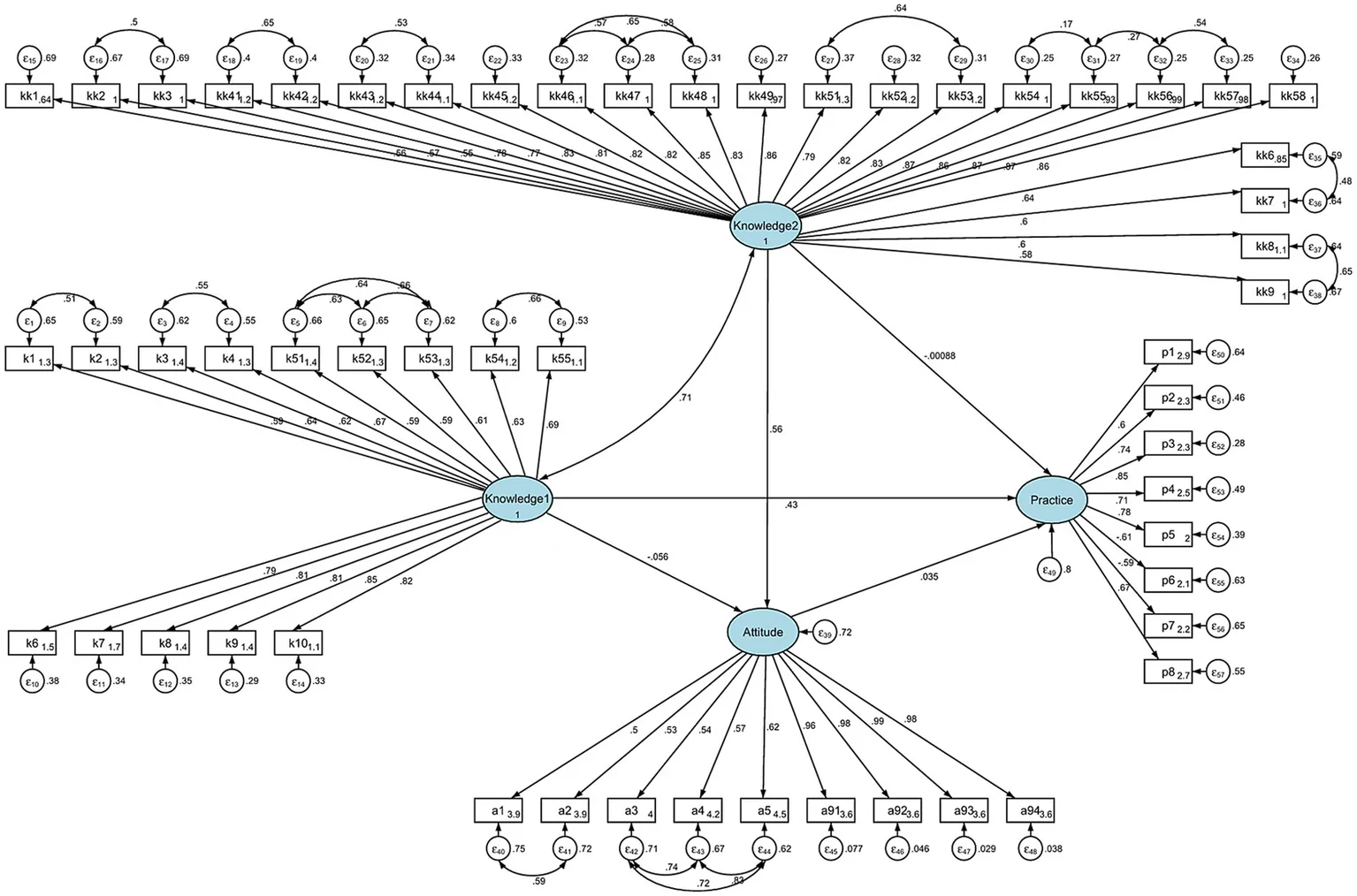
SEM.

The data presented in Supplementary Table 4 indicate significant positive correlations among knowledge, attitudes, and practices (All *p* < 0.001). The model fit indices for the SEM are presented in Supplementary Table 5. Similar to the CFA, the model fit indices indicated an acceptable but suboptimal fit (RMSEA = 0.075, CMIN/DF = 3.604, IFI = 0.872, TLI = 0.863, CFI = 0.872, SRMR = 0.1078). The data presented in Table 4 and Figure 1 illustrate the outcomes of a Structural Equation Modeling (SEM) analysis, describing the total, direct, and indirect associations among the study variables, with a primary focus on the associations of knowledge with attitudes and practices. Specifically, regarding attitudes, the analysis reveals a significant positive association between knowledge about DR and attitude (0.565, 95% CI [0.454, 0.675], *p* < 0.001). Conversely, knowledge about cataracts was not significantly associated with attitude (−0.056, 95% CI [−0.177, 0.066], *p* = 0.369). In the context of practice, the study found a significant positive association between knowledge regarding cataracts and practice (0.428, 95% CI [0.298, 0.558], *p* < 0.001), whereas knowledge concerning DR was not significantly associated with practice (0.019, 95% CI [−0.115, 0.153], *p* = 0.780). Within the practice model pathway, the total association (*β* = 0.428, 95% CI: 0.298 to 0.558, *p* < 0.001) and the direct association (*β* = 0.430, 95% CI: 0.300 to 0.560, *p* < 0.001) between cataract knowledge and practice were statistically significant, whereas the indirect association through attitude was not statistically significant (*β* = −0.002, 95% CI: −0.009 to 0.005, *p* = 0.605). Conversely, the total association (*β* = 0.019, 95% CI: −0.115 to 0.153, *p* = 0.780), direct association (*β* = −0.001, 95% CI: −0.149 to 0.147, *p* = 0.991), and indirect association through attitude (*β* = 0.020, 95% CI: −0.040 to 0.080, *p* = 0.515) between DR knowledge and practice were not statistically significant.

**Table 4:** Interaction among knowledge, attitude, and practice according to SEM.

Model paths	Standardized total effects* (95% CI)	*p*	Standardized direct effects* (95% CI)	*p*	Standardized indirect effects* (95% CI)	*p*
Cataract knowledge → Attitude	−0.028 (−0.196–0.122)	0.713	−0.028 (−0.196–0.122)	0.713		
Cataract knowledge → Practice	−0.027 (0.300–0.626)	0.003	0.436 (0.298–0.613)	0.004	−0.001 (−0.020–0.004)	0.537
Attitude → Practice	0.030 (−0.080–0.124)	0.573	0.030 (−0.080–0.124)	0.573		
DR knowledge → Attitude	0.517 (0.396–0.651)	0.006	0.517 (0.396–0.651)	0.006		
DR knowledge → Practice	0.018 (−0.146–0.204)	0.812	0.003 (−0.168–0.184)	0.979	0.015 (−0.039–0.076)	0.539

*The effects indicate statistical associations rather than causality.

In the univariate linear regression analyses, cataract knowledge score, DR knowledge score, attitude score, age, sex, family history of diabetes, family history of cataract, blood glucose status, and blood lipid status were significantly associated with practice scores. Education level, monthly income, diabetes status, cataract status, and BMI were not significantly associated with practice scores in the univariate analyses. In the multivariable linear regression analysis, higher cataract knowledge scores were independently associated with higher practice scores (*β* = 0.115, 95% CI: 0.034 to 0.197, *p* = 0.034). Higher attitude scores were also positively associated with practice scores (*β* = 0.111, 95% CI: 0.044 to 0.178, *p* = 0.001). Compared with female participants, male participants had lower practice scores (*β* = −1.101, 95% CI: −1.996 to −0.205, *p* = 0.016). Participants who consistently maintained blood lipid levels within the normal range had higher practice scores than those who did not (*β* = 2.101, 95% CI: 1.143 to 3.059, *p* < 0.001). By contrast, DR knowledge score, age, family history of diabetes, family history of cataract, and blood glucose status were not significantly associated with practice scores in the multivariable model. The complete results of the univariate and multivariable linear regression analyses are presented in Supplementary Table 6.

## Discussion

The analysis of 460 valid survey samples reveals a limited understanding of two prevalent eye diseases, cataract and DR, among the population. Furthermore, among the participants in this rural community, greater awareness of cataract and DR was associated with more positive attitudes and better health-related practices. These findings may have practical implications for the development of targeted health education initiatives in rural communities. In addition, female participants accounted for a higher proportion of the sample than male participants (62.0% vs. 38.0%). The multivariable analysis showed that male participants had lower practice scores than female participants after adjustment for other included factors. Therefore, both the sex-related difference in practice scores and the unequal sex distribution should be considered when interpreting the overall practice findings.

This study found that most people lack awareness about blinding eye diseases, such as DR and cataracts, despite their prevalence. In remote areas, limited health education worsens this issue, and the subtle early symptoms of DR lead to delayed recognition, reducing awareness of its severity (12, 24, 25). Residents’ understanding of cataracts and DR is closely associated with their attitudes and actions. Lack of knowledge has been associated with delayed medical help-seeking and poorer disease management (26, 27). In rural areas, limited medical resources, such as eye exam equipment and specialists, hinder timely diagnosis and treatment (17). The mechanisms underlying knowledge deficiency are multifaceted. Lower educational attainment is associated with lower health literacy and poorer comprehension of medical information (11, 19). Economic constraints limit access to healthcare services and health education resources (17). Additionally, traditional beliefs and misconceptions about eye diseases may impede acceptance of scientific knowledge (26). The lack of accessible information channels in rural areas further exacerbates this knowledge gap. From the specific knowledge items, awareness was particularly low regarding the diagnostic criteria for DR (29.3%) and the classification of DR into non-proliferative and proliferative stages (37.2%). Similarly, less than one-third of participants were knowledgeable about cataract diagnostic methods (24.6%) and the typical symptoms of cataracts (27.8%). These findings suggest that participants had limited understanding of disease definition, early identification, and clinical staging, which may be associated with delayed screening and treatment-seeking behavior.

In this study, greater knowledge of DR was positively associated with more favorable attitudes, whereas cataract knowledge was positively associated with practice. The association between DR knowledge and attitude may be related to participants’ awareness of diabetes-related complications (12, 24, 25). In contrast, the association between cataract knowledge and practice may reflect the relatively clear screening and treatment pathway for cataract, which may facilitate appropriate eye-care and healthcare-seeking behavior (19). However, DR knowledge was not significantly associated with practice, possibly because DR prevention and management require multiple sustained behaviors, including glycemic control, regular fundus examinations, medication adherence, and lifestyle modification (28). By comparison, cataract management is more procedure-oriented, and once individuals understand that surgery is effective, knowledge may more readily translate into concrete healthcare-seeking behavior. It should be emphasized that surgery is the established definitive treatment for cataracts. Although pharmacological approaches may be used to manage certain symptoms or coexisting ocular conditions, they do not reverse cataract-related lens opacity (3). The multivariable linear regression analysis further showed that cataract knowledge, attitude, sex, and blood lipid status were independently associated with practice scores. The positive associations of cataract knowledge and attitude with practice are consistent with the KAP framework, indicating that greater disease-related knowledge and more favorable attitudes may support the adoption of appropriate eye-care behaviors. Male participants had lower practice scores than female participants, which may reflect differences in preventive healthcare utilization, health-information seeking, or willingness to seek medical advice. Participants who consistently maintained blood lipid levels within the normal range also had higher practice scores, suggesting that better general health-management behaviors may coexist with more appropriate eye-health practices. However, these associations should be interpreted cautiously because the cross-sectional design does not permit causal inference.

This study has several limitations. First, the recruitment method using QR code-based, self-administered surveys may have introduced selection bias. Given the older target population (ages 40–80), individuals lacking technical literacy, internet access, or adequate vision may have been unable to participate through the digital platform. As a result, our sample may over-represent more educated and technologically capable individuals, potentially limiting the representativeness of the findings to the broader rural older adults. Therefore, the findings may underestimate the knowledge and practice gaps among older rural residents with limited digital literacy or access to digital devices. In addition, the higher proportion of female participants and the observed gender difference in practice scores may have influenced the overall practice score estimates, further limiting the representativeness of the findings. Second, this was a single-region study conducted among middle-aged and older adults residing in Huanggang town, Shanxian county, Shandong province. Local socioeconomic conditions, healthcare accessibility, educational resources, and patterns of health information dissemination may differ from those in other rural regions. Therefore, the findings may not be generalizable to rural populations in other regions of China or to the broader middle-aged and older population. Future multicenter studies involving geographically and socioeconomically diverse populations are needed to validate and extend these findings. Third, 33.5% of the participants had received only primary school education or below. Although trained field workers were available to clarify questions when necessary, some participants may still have had difficulty understanding certain medical terms or questionnaire items, which could have introduced response bias or misinterpretation and affected the accuracy of the findings. Fourth, the survey did not explore how the participants obtain information, limiting authorities’ ability to improve information dissemination. In addition, although multivariable linear regression was performed, residual confounding from unmeasured or incompletely adjusted factors cannot be excluded. Finally, the structural validity of the measurement model (results for CFA and SEM) needs to be explained with caution. Although most fit indices indicated an acceptable model fit, the standardized root mean square residual (SRMR) value marginally exceeded the strict threshold of 0.08. Although fit indices may be penalized in finite samples with a larger number of observed variables (29, 30), the overall fit should be regarded as acceptable but suboptimal rather than good. The CFA and SEM results should therefore be interpreted with caution, and future studies with refined measurement models are warranted. Furthermore, because the content validity of the questionnaire was only qualitatively assessed, future studies should incorporate rigorous quantitative validation of the survey instrument.

Both conditions can be prevented and managed. Early detection of cataracts allows for timely surgery, while managing DR largely depends on the patient’s self-care. Additionally, future studies should develop and evaluate health education interventions for rural communities, focusing on improving awareness of cataract and DR among middle-aged and older adults and examining whether such interventions are associated with earlier diagnosis, timely treatment, and improved eye-health outcomes. Because no intervention data were collected in this study, the following are presented as potential implications rather than evidence-based recommendations: (1) establishing community-based health education programs with simplified visual materials suitable for old adults populations; (2) implementing regular eye screening programs in rural areas through mobile medical units; (3) providing subsidies for cataract surgery and DR treatment to reduce financial barriers; (4) training village healthcare workers to deliver basic eye health education; and (5) leveraging community leaders and interpersonal networks for health information dissemination.

## Data Availability

The original contributions presented in the study are included in the article/Supplementary material, further inquiries can be directed to the corresponding author.
