# Supplementary material for: Knowledge, attitudes, and practices regarding cataract and diabetic retinopathy among middle-aged population and older adults in a rural area at eastern China
Source: Front Public Health. 2026 Sep 1;14:1888283. doi: 10.3389/fpubh.2026.1888283 (PMC13574652; doi:10.3389/fpubh.2026.1888283)
Supplement: Supplementary file 1 [file Supplementary_file_1.docx]

Dear Participant,

We sincerely invite you to participate in our research study. This study aims to understand the knowledge, attitudes, and practices of middle-aged and elderly individuals in rural areas regarding cataracts and diabetic retinopathy. The findings will provide a basis for developing scientific intervention strategies that may help improve the health of more individuals in the future. Your participation in this research is voluntary. If you agree to participate, please refer to the following instructions.

1. Please complete the questionnaire. There are no right or wrong answers. You only need to fill in according to the actual situation. You can ask us any questions during the answering process. Please submit it promptly after completion.

2. This study is a simple questionnaire survey, which will not cause harm to your physical and mental health. However, it will involve some privacy issues, such as your gender, age, etc. We will strictly protect your confidentiality and will not disclose your information. Please feel free to fill it out.

3. As a participant, you can always understand the information and research progress related to this study. If you decide to withdraw from the study, please inform us. Your data will not be included in the research results.

Finally, we sincerely thank you for taking the time to support our scientific research amidst your busy schedule!

□ I am aware of and agree to the use of the collected data for scientific research.

Informed Consent Signature:

Date of Participation: Year	Month	Day

| **Part I: Basic Information** | |
| --- | --- |
| **1.Your age** | Years |
| **2.Your gender:** | a.Male  b.Famale |
| **3.Your education level:** | a. Primary school and below  b. Junior high school/high school/vocational school  c. College/undergraduate  d. Master's degree and above |
| **4.In the past year, your household's monthly per capita income (including income from in-kind benefits and rental income, etc.): ______ yuan** | a.<5000  b.5000-10000  c.10000-20000  d.>20000 |
| **5.Do you have diabetes?** | a.Yes  b.No |
| **5-1.what type of diabetes do you have? (to be answered by participants with diabetes)** | a. Type I diabetes  b. Type II diabetes  c.Other types  d.Unsure |
| **5-2.How long have you been diagnosed with diabetes?**  **(to be answered by participants with diabetes)** | Years |
| **5-3.Have you ever undergone a fundus examination?**  **(to be answered by participants with diabetes)** | a.Yes  b.No |
| **5-4.Have you been diagnosed with diabetic retinopathy?**  **(to be answered by participants with diabetes)** | a.Yes  b.No |
| **6.Do you have a family history of diabetes?** | a.Yes  b.No  c.Unsure |
| **6-1.Do you have a habit of consuming a highsugar diet?**  **(to be answered by participants with diabetes)** | a.Yes  b.No |
| **6-2.Are you obese?**  **(to be answered by participants with diabetes)** | a.Yes  b.No |
| **6-3.Do you regularly undergo glycated haemoglobin and blood glucose testing?**  **(to be answered by participants with diabetes)** | a.Yes  b.No |
| 7.**Do you have cataracts?** | a.Yes  b.No |
| **7-1.What type of cataracts do you have?**  **(Participants with cataracts are to answer):** | a.Congenital cataracts  b.Senile cataracts  c.Other types  d.Unsure |
| **7-2.How long have you had cataracts?**  **(Participants with cataracts are to answer):** | Years |
| **8.Do you have a family history of cataracts?** | a.Yes  b.No  c.Unsure |
| **9.Your height:** | cm |
| **10.Your weight:** | kg |
| **11. Is your blood glucose level within the normal range?** | a. Always (never experiences abnormalities)  b. Occasionally experiences abnormalities  c. Frequently experiences abnormalities  d. Uncertain |
| **12.Is your blood lipid level within the normal range?** | a. Always (never experiences abnormalities)  b. Occasionally experiences abnormalities  c. Frequently experiences abnormalities  d. Uncertain |
| **13.** **Do you have cataracts?** | a.Yes  b.No  c.Unsure |

**Part II-Knowledge (Cataract)**

**Please select one option from "Understand," "Heard of," or "Unfamiliar" based on your knowledge of the relevant descriptions. If you fully understand the statement, please choose "Understand"; if you have heard of the relevant description but are unclear about the specific content, please choose "Heard of."**

| 1. Are you aware of the diagnostic methods for cataracts? | **a.**Knowledgeable | **b.**Heard of It | **c.**Unaware |
| --- | --- | --- | --- |
| 2. Cataracts can be classified as congenital or acquired, with senile cataracts being the most common type among acquired cataracts. | **a.**Knowledgeable | **b.**Heard of It | **c.**Unaware |
| 3. The most typical symptom of cataracts is a gradual, painless decline in vision. Other symptoms include blurred vision and decreased colour perception. | **a.**Knowledgeable | **b.**Heard of It | **c.**Unaware |
| 4. Early-stage cataracts have inconspicuous symptoms and can be easily confused with presbyopia or visual fatigue. | **a.**Knowledgeable | **b.**Heard of It | **c.**Unaware |
| 5. Are you aware that the occurrence of cataracts may be related to the following factors? |  |  |  |
| Advanced age, glaucoma, high myopia, metabolic diseases (e.g., diabetes) | **a.**Knowledgeable | **b.**Heard of It | **c.**Unaware |
| Intense light exposure, radiation exposure, hightemperature working environment | **a.**Knowledgeable | **b.**Heard of It | **c.**Unaware |
| History of ocular trauma, inflammation, or surgery | **a.**Knowledgeable | **b.**Heard of It | **c.**Unaware |
| Poor lifestyle habits (e.g., smoking, excessive alcohol consumption, obesity) | **a.**Knowledgeable | **b.**Heard of It | **c.**Unaware |
| Long-term use of medications such as glucocorticoids or miotic agents | **a.**Knowledgeable | **b.**Heard of It | **c.**Unaware |
| 6. If cataracts are not treated promptly, secondary glaucoma, visual impairment, or even blindness may occur. | **a.**Knowledgeable | **b.**Heard of It | **c.**Unaware |
| 7. Treatment for cataracts includes pharmacological and surgical interventions, with surgery being the primary treatment option. | **a.**Knowledgeable | **b.**Heard of It | **c.**Unaware |
| 8. If the patient has both diabetes and cataracts, it is necessary to stabilise blood glucose levels and perform a comprehensive fundus examination before surgical treatment. | **a.**Knowledgeable | **b.**Heard of It | **c.**Unaware |
| 9. Surgical treatment of cataracts may lead to complications such as infection, inflammatory reactions, and dry eye syndrome. | **a.**Knowledgeable | **b.**Heard of It | **c.**Unaware |
| 10. Post-surgical outcomes indicate that most patients achieve a corrected distance visual acuity of 1.0 or higher. | **a.**Knowledgeable | **b.**Heard of It | **c.**Unaware |

**Part II-Knowledge (Diabetic Eye Disease)**

| 1. Do you understand the diagnostic criteria for diabetic retinopathy (DR)? | **a.**Yes | **b.**No |  |
| --- | --- | --- | --- |
| 2. DR is one of the complications of diabetes. | **a.**Yes | **b.**No | **c.**Unsure |
| 3. Patients with diabetes may develop DR even if their blood glucose levels are well controlled. | **a.**Yes | **b.**No | **c.**Unsure |
| 4. Are you aware that the onset of DR may be related to the following factors? |  |  |  |
| - Age at diabetes onset | **a.**Yes | **b.**No | **c.**Unsure |
| - Fasting blood glucose levels | **a.**Yes | **b.**No | **c.**Unsure |
| - Glycated haemoglobin levels | **a.**Yes | **b.**No | **c.**Unsure |
| - Hypertension | **a.**Yes | **b.**No | **c.**Unsure |
| - Hyperlipidaemia | **a.**Yes | **b.**No | **c.**Unsure |
| - Smoking | **a.**Yes | **b.**No | **c.**Unsure |
| - Poor treatment adherence | **a.**Yes | **b.**No | **c.**Unsure |
| - Alcohol consumption | **a.**Yes | **b.**No | **c.**Unsure |
| - Renal function | **a.**Yes | **b.**No | **c.**Unsure |
| 5. Are you aware of the following symptoms of DR? |  |  |  |
| - Decreased vision | **a.**Yes | **b.**No | **c.**Unsure |
| - Flashing lights or floaters | **a.**Yes | **b.**No | **c.**Unsure |
| - Blurred vision (or distorted images) | **a.**Yes | **b.**No | **c.**Unsure |
| - Visual field loss | **a.**Yes | **b.**No | **c.**Unsure |
| - Double vision | **a.**Yes | **b.**No | **c.**Unsure |
| - Darkening or fading of colours | **a.**Yes | **b.**No | **c.**Unsure |
| - Eye pain | **a.**Yes | **b.**No | **c.**Unsure |
| - Blindness | **a.**Yes | **b.**No | **c.**Unsure |
| 6. Are you aware that DR can be classified into non-proliferative and proliferative forms based on severity? | **a.**Yes | **b.**No | **c.**Unsure |
| 7. Are you aware that patients with diabetes need to undergo a fundus examination upon diagnosis and regularly receive follow-up assessments? | **a.**Yes | **b.**No | **c.**Unsure |
| 8. Are you aware that DR can be treated with medications or surgical interventions? | **a.**Yes | **b.**No | **c.**Unsure |
| 9. Are you aware that DR can also be managed through non-pharmacological means such as diet and exercise? | **a.**Yes | **b.**No | **c.**Unsure |

**Part-III Attitudes**

**Please select one option ranging from "Strongly Agree" to "Strongly Disagree" based on your level of agreement with the statement in the question.**

| 1. Cataracts and diabetic retinopathy (DR) can affect vision and may lead to blindness in severe cases. | a.Strongly Agree | b.Agree | c.Neutral | d.Disagree | e.Strongly Disagree |
| --- | --- | --- | --- | --- | --- |
| 2. My own actions are key to the prevention and management of cataracts and DR. | a.Strongly Agree | b.Agree | c.Neutral | d.Disagree | e.Strongly Disagree |
| 3. Even if my vision is normal, I need to undergo regular vision and fundus examinations. | a.Strongly Agree | b.Agree | c.Neutral | d.Disagree | e.Strongly Disagree |
| 4. Proper dietary control is crucial for the prevention (or treatment) of cataracts and DR. | a.Strongly Agree | b.Agree | c.Neutral | d.Disagree | e.Strongly Disagree |
| 5. Changing unhealthy lifestyle habits (such as smoking, drinking alcohol, and overeating) is essential for the prevention (or treatment) of cataracts and DR. | a.Strongly Agree | b.Agree | c.Neutral | d.Disagree | e.Strongly Disagree |
| 6. I worry that even surgical treatment may not restore my vision to normal. | a.Strongly Agree | b.Agree | c.Neutral | d.Disagree | e.Strongly Disagree |
| 7. I am concerned about potential complications associated with surgery. | a.Strongly Agree | b.Agree | c.Neutral | d.Disagree | e.Strongly Disagree |
| 8. I fear the risk of blindness following surgery. | a.Strongly Agree | b.Agree | c.Neutral | d.Disagree | e.Strongly Disagree |
| 9. Regarding the prevention and management of cataracts and DR, would you like assistance in the following areas? |  |  |  |  |  |
| - Regular fundus and vision examinations | a.Strongly Agree | b.Agree | c.Neutral | d.Disagree | e.Strongly Disagree |
| - Disease-related education | a.Strongly Agree | b.Agree | c.Neutral | d.Disagree | e.Strongly Disagree |
| - Dietary and treatment guidance | a.Strongly Agree | b.Agree | c.Neutral | d.Disagree | e.Strongly Disagree |
| - Health knowledge seminars | a.Strongly Agree | b.Agree | c.Neutral | d.Disagree | e.Strongly Disagree |

**Part IV- Practice**

**Please select the option that best reflects your situation based on the choices you made in the corresponding circumstances.**

| 1. You ensure that blood glucose/lipid levels remain within normal ranges by controlling your diet. | b.Often | c.Sometimes | d.Occasionally | e.Never |
| --- | --- | --- | --- | --- |
| 2. You ensure that blood glucose/lipid levels remain within normal ranges through the use of medications. | b.Often | c.Sometimes | d.Occasionally | e.Never |
| 3. You undergo regular vision and fundus examinations. | b.Often | c.Sometimes | d.Occasionally | e.Never |
| 4. You are able to change unhealthy lifestyle habits, such as smoking and drinking alcohol. | b.Often | c.Sometimes | d.Occasionally | e.Never |
| 5. Do you protect your eyes during outdoor activities by wearing sunglasses or similar protective measures? | b.Often | c.Sometimes | d.Occasionally | e.Never |
| 6. Do you often experience eye strain? | b.Often | c.Sometimes | d.Occasionally | e.Never |
| 7. Do you use medications without consulting a doctor? | b.Often | c.Sometimes | d.Occasionally | e.Never |
| 8. When experiencing eye symptoms, do you contact a doctor immediately? | b.Often | c.Sometimes | d.Occasionally | e.Never |
