# Supplementary material for: Knowledge, attitudes, and practices regarding cataract and diabetic retinopathy among middle-aged population and older adults in a rural area at eastern China
Source: Front Public Health. 2026 Sep 1;14:1888283. doi: 10.3389/fpubh.2026.1888283 (PMC13574652; doi:10.3389/fpubh.2026.1888283)
Supplement: Supplementary file 2 [file Supplementary_file_2.docx]

**Supplementary Table 1. Model Fit Indices for the Confirmatory Factor Analysis**

|  | **Ref.** | **Measured results** |
| --- | --- | --- |
| **RMSEA** | <0.080 | 0.080 |
| **SRMR** | <0.080 | 0.1046 |
| **CMIN/DF** | 1-3 excellent, 3-5 good | 3.914 |
| **IFI** | >0.8 good | 0.856 |
| **TLI** | >0.8 good | 0.847 |
| **CFI** | >0.8 good | 0.856 |

**Supplementary Table 2 Response to Attitudes**

| **Attitude** | **Strongly Agree** | **Agree** | **Neutral** | **Disagree** | **Strongly Disagree** |
| --- | --- | --- | --- | --- | --- |
| 1. Cataracts and diabetic retinopathy (DR) can affect vision and may lead to blindness in severe cases. | 182(39.6%) | 167(36.3%) | 74(16.1%) | 19(4.1%) | 18(3.9%) |
| 2. My own actions are key to the prevention and management of cataracts and DR. | 168(36.5%) | 178(38.7%) | 74(16.1%) | 25(5.4%) | 15(3.3%) |
| 3. Even if my vision is normal, I need to undergo regular vision and fundus examinations. | 174(37.8%) | 179(38.9%) | 69(15%) | 24(5.2%) | 14(3%) |
| 4. Proper dietary control is crucial for the prevention (or treatment) of cataracts and DR. | 177(38.5%) | 189(41.1%) | 59(12.8%) | 24(5.2%) | 11(2.4%) |
| 5. Changing unhealthy lifestyle habits (such as smoking, drinking alcohol, and overeating) is essential for the prevention (or treatment) of cataracts and DR. | 190(41.3%) | 187(40.7%) | 55(12%) | 18(3.9%) | 10(2.2%) |
| 6. I worry that even surgical treatment may not restore my vision to normal. | 19(4.1%) | 22(4.8%) | 4(0.9%) | 3(0.7%) | 2(0.4%) |
| 7. I am concerned about potential complications associated with surgery. | 18(3.9%) | 27(5.9%) | 2(0.4%) | 0 (0%) | 3(0.7%) |
| 8. I fear the risk of blindness following surgery. | 18(3.9%) | 26(5.7%) | 3(0.7%) | 2(0.4%) | 1(0.2%) |
| 9. Regarding the prevention and management of cataracts and DR, would you like assistance in the following areas? |  |  |  |  |  |
| - Regular fundus and vision examinations | 263(57.2%) | 106(23%) | 47(10.2%) | 9(2%) | 35(7.6%) |
| - Disease-related education | 248(53.9%) | 118(25.7%) | 51(11.1%) | 10(2.2%) | 33(7.2%) |
| - Dietary and treatment guidance | 245(53.3%) | 124(27%) | 51(11.1%) | 7(1.5%) | 33(7.2%) |
| - Health knowledge seminars | 253(55%) | 114(24.8%) | 50(10.9%) | 8(1.7%) | 35(7.6%) |

**Supplementary Table 3. Response to Practice**

| **Practice** | **Always** | **Often** | **Sometimes** | **Occasionally** | **Never** |
| --- | --- | --- | --- | --- | --- |
| 1. You ensure that blood glucose/lipid levels remain within normal ranges by controlling your diet. | 166(36.1%) | 107(23.3%) | 108(23.5%) | 38(8.3%) | 41(8.9%) |
| 2. You ensure that blood glucose/lipid levels remain within normal ranges through the use of medications. | 138(30%) | 92(20%) | 108(23.5%) | 34(7.4%) | 88(19.1%) |
| 3. You undergo regular vision and fundus examinations. | 101(22%) | 78(17%) | 115(25%) | 94(20.4%) | 72(15.7%) |
| 4. You are able to change unhealthy lifestyle habits, such as smoking and drinking alcohol. | 152(33%) | 80(17.4%) | 113(24.6%) | 52(11.3%) | 63(13.7%) |
| 5. Do you protect your eyes during outdoor activities by wearing sunglasses or similar protective measures? | 111(24.1%) | 80(17.4%) | 88(19.1%) | 76(16.5%) | 105(22.8%) |
| 6. Do you often experience eye strain? | 95(20.7%) | 118(25.7%) | 110(23.9%) | 83(18%) | 54(11.7%) |
| 7. Do you use medications without consulting a doctor? | 83(18%) | 69(15%) | 103(22.4%) | 95(20.7%) | 110(23.9%) |
| 8. When experiencing eye symptoms, do you contact a doctor immediately? | 134(29.1%) | 107(23.3%) | 107(23.3%) | 74(16.1%) | 38(8.3%) |

**Supplementary Table 4. Correlation Analysis**

| **Spearman** | **Knowledge on cataract** | **Knowledge on DR** | **Knowledge (cataract + DR)** | **Attitudes** | **Practice** |
| --- | --- | --- | --- | --- | --- |
| Knowledge on cataract | 1.000 |  |  |  |  |
| Knowledge on DR | 0.742 (<0.001) | 1.000 |  |  |  |
| Knowledge (cataract + DR) | 0.866 (<0.001) | 0.972 (<0.001) | 1.000 |  |  |
| Attitudes | 0.504 (<0.001) | 0.601 (<0.001) | 0.607 (<0.001) | 1.000 |  |
| Practice | 0.381 (<0.001) | 0.372 (<0.001) | 0.390 (<0.001) | 0.322 (<0.001) | 1.000 |

**Supplementary Table 5. Model Fit Indices for the Structural Equation Model**

|  | **Ref.** | **Measured results** |
| --- | --- | --- |
| **RMSEA** | <0.080 | 0.075 |
| **SRMR** | <0.080 | 0.1078 |
| **CMIN/DF** | 1-3 excellent, 3-5 good | 3.604 |
| **IFI** | >0.8 good | 0.872 |
| **TLI** | >0.8 good | 0.863 |
| **CFI** | >0.8 good | 0.872 |

**Supplementary Table 6. Univariate and Multivariable Linear Regression Analyses of Factors Associated with Practice Scores**

| Practice | Univariate liner regression | | Multivariate liner regression | |
| --- | --- | --- | --- | --- |
|  | β (95%CI) | P | β (95%CI) | P |
| Cataract knowledge score | 0.251(0.195- 0.306) | <0.001 | 0.115(0.034-0.197) | 0.034 |
| DR knowledge score | 0.105(0.079-0.131) | <0.001 | 0.019(-0.020-0.058) | 0.348 |
| Attitude score | 0.204(0.147- 0.262) | <0.001 | 0.111(0.044-0.178) | 0.001 |
| Age | -0.093(-0.150- -0.035) | 0.002 | -0.022(-0.079-0.035) | 0.450 |
| Gender |  |  |  |  |
| Male | -1.415(-2.414- -0.416) | 0.006 | -1.101(-1.996- -0.205) | 0.016 |
| Female | ref |  |  |  |
| **Education** |  |  |  |  |
| Primary school or below | -0.999(-2.287-0.288) | 0.128 |  |  |
| Secondary school/High school/Vocational school | -0.729(-1.963-0.505) | 0.246 |  |  |
| College or above | ref |  |  |  |
| **Monthly income, CNY** |  |  |  |  |
| < 5000 | ref |  |  |  |
| 5000-10000 | 0.123(-1.057-1.304) | 0.837 |  |  |
| > 10000 | 0.318(-1.391-2.026) | 0.715 |  |  |
| **Diabetes** |  |  |  |  |
| Yes | -1.352(-2.994-0.290) | 0.106 |  |  |
| No | ref |  |  |  |
| **Family history of diabetes** |  |  |  |  |
| Yes | 1.049(-1.090-3.188) | 0.336 | -0.089(-2.063-1.885) | 0.929 |
| No | 2.113(0.543-3.683) | 0.008 | 0.054(-1.544-1.652) | 0.067 |
| Unsure | ref |  | ref |  |
| **Cataracts** |  |  |  |  |
| Yes | -1.301(-2.868-0.266) | 0.103 |  |  |
| No | ref |  |  |  |
| **Family history of cataracts** |  |  |  |  |
| Yes | 1.515(-0.748-3.778) | 0.189 | 0.516(-1.621-2.652) | 0.635 |
| No | 2.898(1.663-4.134) | <0.001 | 1.327(-0.041-2.695) | 0.057 |
| Unsure | ref |  | Ref |  |
| **BMI, kg/m^2^** |  |  |  |  |
| < 24 | 0.705(-0.273-1.683) | 0.157 |  |  |
| ≥ 24 | ref |  |  |  |
| **Blood glucose within the normal range** |  |  |  |  |
| Always | 3.381(2.448-4.314) | <0.001 | -0.141(-1.706-1.425) | 0.860 |
| Other | ref |  | ref |  |
| **Blood lipid within the normal range** |  |  |  |  |
| Always | 3.208(2.240-4.176) | <0.001 | 2.101(1.143-3.059) | <0.001 |
| Other | ref |  |  |  |
